# The health service perspective on determinants of success in allied health student research project collaborations: a qualitative study guided by the Consolidated Framework for Implementation Research

**DOI:** 10.1186/s12913-024-10599-8

**Published:** 2024-01-27

**Authors:** Rebecca L. Angus, H. Laetitia Hattingh, Kelly A. Weir

**Affiliations:** 1 Allied Health and Rehabilitation Services, Gold Coast Hospital and Health Service, 1 Hospital Boulevard, Southport, QLD 4215 Australia; 2 Medical Services, Clinical Governance and Research, Gold Coast Hospital and Health Service, 1 Hospital Boulevard, Southport, QLD 4215 Australia; 3 School of Health Sciences and Social Work, Griffith University, Gold Coast, QLD Australia

**Keywords:** Research capacity building, Allied health, Healthcare performance, Research supervision, CFIR

## Abstract

**Background:**

A research culture in health care organisations is associated with improved healthcare performance. Allied health (AH) students undertake research training as part of their professional degree qualifications. This may include participation in research projects, sometimes undertaken in association with health services. Co-supervision of these projects by health service staff provides research capacity building opportunities and staff-centred outcomes for the individuals involved, as well as improvements in clinical knowledge and practice within the local area. Also, publications from these projects contribute to the wider evidence base. Identification of barriers and facilitators to engagement in, and conduct of, these projects may optimise systems for improved health service outcomes.

**Methods:**

This formative evaluation used the Consolidated Framework for Implementation Research (CFIR) to guide analysis of qualitative data obtained from semi-structured interviews with health service-employed allied health professionals, including clinicians and research fellows, who had supervised students on clinical-related research placements within the previous five years.

**Results:**

Eleven AH clinicians described 18 collaborative projects with 24 students from five AH disciplines across four universities. Three health service-employed AH research fellows described their involvement in these and other student research projects. Twenty key determinant constructs were identified and mapped across all five CFIR domains. Facilitators included health service cosmopolitanism, project adaptability and implementation climate (compatibility). Health service-employed research fellows provided readiness for implementation and a facilitator for project execution. The main barriers identified were cost to staff in workload and personal time and aspects related to project complexity. Differing student characteristics affected the relative advantage of collaborative projects in positive and negative manners.

**Conclusions:**

This study describes the facilitators and barriers to the conduct of collaborative AH student research projects. Addressing these determinants when establishing each new project may enable health services to optimise communication, role delineation and project success, and thus ultimately, healthcare performance and patient care.

**Supplementary Information:**

The online version contains supplementary material available at 10.1186/s12913-024-10599-8.

## Background

The value of a research culture within health services is well recognised, with improvements in healthcare performance associated with engagement of these organisations and their staff in research [1]. Health service based research has relevance to practice, and research engaged clinicians are likely to adopt new evidence-based practices, which is particularly important in fields where the science is rapidly changing [1]. The resulting organisational benefits include improved staff recruitment and retention, improved care and delivery pathways, wider access to evidence-based healthcare, better patient/carer experience and reduced patient mortality [2, 3].

Allied health (AH) professionals include university qualified practitioners with specialised expertise in preventing, diagnosing and treating a range of conditions and illnesses. AH encompasses a range of different disciplines excluding medicine, nursing and dentistry [4]. Making up a large and growing proportion of the health workforce, in Australia, AH professionals constitute over one quarter of the health workforce [5]. As such, building the research potential of AH professionals is an essential component of improving overall healthcare performance. Research awareness is considered an entry level skill for AH professionals, as practice within an evidence-based paradigm is a core principle [4, 6]. In addition to coursework-based research training, the professional degree programs of many AH disciplines offer participation in supervised research projects. Projects may be university based, but in some cases are conducted in collaboration with external organisations, including healthcare institutions. As well as providing benefits for students, involvement in these collaborative student research projects can also have valuable outcomes for health services and their staff [7–9]. Our previous work [7] indicated this includes wide ranging impacts on healthcare through knowledge translation, as well as improvements at a local level through increased clinical practice knowledge of staff and provision of evidence to support current processes, or practice change improvements. Research capacity gains include increased research knowledge and skills, the formation or affirmation of collaborations, and opportunities for future research. Staff-centred outcomes including job satisfaction were also evident. However, the experiences of health service clinician supervisors sometimes fell short of their expectations [7]. Thus, there is potential for improving the management of these collaborative student research projects. Factors affecting project initiation and progress, particularly those identified from the health service perspective and thus within immediate sphere of influence, may provide points to address for optimisation of the benefits for health services.

Conceptual frameworks provide a guide for the systematic assessment of factors that influence the enactment of new initiatives, and the effectiveness of implementing these within complex systems such as healthcare organisations. Application provides unifying terminology to enable meaningful comparison and contrast between different studies and supports generalisation of results between contexts. Frameworks may also aid in the identification of determinants that may be overlooked during inductive analysis alone. One of the most highly cited determinant frameworks is the Consolidated Framework for Implementation Research (CFIR) [10]. The CFIR is comprised of 39 constructs organised within five domains (innovation characteristics, outer setting, inner setting, characteristics of the individuals involved, and the process of implementation) and was designed to be used flexibly to accommodate the specific context of a study [11]. The CFIR can be used at any implementation phase, from pre-implementation planning to post-implementation evaluations of effectiveness. It can also be used in real-time assessment of implementation progress, to inform strategies for improvement. In this study, we used the CFIR in a formative evaluation to assess progress in the implementation of collaborative student research projects within our health service. The aim was to identify determinants (barriers and facilitators) to engagement in, and conduct of, these projects from the health service perspective, to enable future systems optimisation for improved health service outcomes.

## Methods

The CFIR was used to guide analysis of qualitative data obtained from semi-structured interviews with health service employed AH professionals who had supervised students on clinical-related research placements within the previous five years. Projects were undertaken as part of students’ professional degree qualification programs (Bachelor or Master professional qualification, excluding students undertaking a higher degree by research (HDR)). Staff from all allied health disciplines in our health service were eligible for participation (audiology, dietetics, occupational therapy, pharmacy, physiotherapy, podiatry, psychology, social work or speech pathology). This study was approved by the Gold Coast Hospital and Health Service Human Research Ethics Committee. The manuscript follows the consolidated criteria for reporting qualitative research [12].

### Setting and researcher positionality

The study was undertaken at a tertiary health service in southeast Queensland, Australia providing publicly funded inpatient and outpatient health services to a local population of 650,000 people. Gold Coast Hospital and Health Service (GCHHS) has an AH workforce of 1200, which includes a small number of research fellows employed to support research capacity building.

State-wide health practitioner role descriptions for all allied health disciplines include provision of clinical practice supervision for students on placement [13]. Clinical placements are managed at the Health Service departmental discipline level on a contract basis with partner universities. In contrast, student research placements are generally undertaken only on an ad hoc basis, as opportunity and staff interest arise.

The multidisciplinary study team comprised three career researchers, all holding research doctorates, professional qualifications and registration in allied health (RA-dietetics, LH-pharmacy, KW-speech pathology), and with experience in qualitative interviewing and analysis methods. All were female and in addition to health service employment held simultaneous conjoint (KW) or adjunct (honorary—RA, LH) appointments with partner universities. RA and LH had previously co-supervised student research project collaborations with clinicians, and all team members had at times worked with health service clinicians to resolve issues arising during or after completion of these student placements. As such, the team took an insider perspective to the study. The research team’s professional backgrounds were known to the participants, and in some cases, a researcher had previously provided support with a participant’s collaborative student research project. For these, interviews were conducted by a team member not involved in that project.

### Data collection

This study explored the perspectives of health service staff with direct involvement in collaborative student research projects on the facilitators and barriers to their successful conduct. Discussion with AH discipline leads, research staff and snowball sampling were used for purposive identification of AH professionals who had supervised students on clinical-related research placements within the previous five years. All participants were over 18 years of age. Recruitment was through direct email approach by the researchers, continuing until no further participants could be identified. A study information sheet was provided and written informed consent collected from those agreeing to participate. A single, semi-structured interview was conducted with each participant, using a guide described in Angus et al. 2022 [7] designed to facilitate free flowing interviews to support both deductive and inductive analysis. The guide was developed with reference to the CFIR [11], and based on a review of the literature and the professional experiences of the research team. Through this, 21 constructs across 4 CFIR domains were identified for questioning (Additional file 1). Participants were advised that interviews were confidential, would not affect their employment and that information would be de-identified for publication. Interviews were face-to-face in a private room with only interviewer and participant present, were conducted between March and July 2021 and lasted 21–58 min. Following interviews, field notes were made to capture non-verbal content and allow contextualisation of data. Demographic information and details of projects, university and student collaborators were captured via brief survey prior to interview. Transcripts of the audio recorded interviews were checked against recordings for accuracy but not returned for member checking.

### Data analysis and interpretation

Within the context of this study, we defined the CFIR domains as: I. Innovation—The act of health service-employed AH professionals supervising or co-supervising students on a clinically relevant research project undertaken as part of students’ professional degree qualification program; II. Outer Setting—The external social and political context including Australian and Queensland government policies and in particular, aspects related to universities in the local area offering AH degree programs, and their students; III. Inner Setting—The employees, departments, systems, policies and resources of a tertiary hospital and health service located in south-east Queensland; IV. Characteristics of Individuals—Those of the people employed within the inner setting, specifically, AH professionals and specialist research staff; V. Process—The process of conducting the collaborative student research project (the innovation) in its entirety from planning, engaging individuals within the inner and outer settings, executing the project and its evaluation.

The CFIR codebook template [11] was annotated with definitions adapted to the study context. The only major change from the CFIR template construct definitions of relevance to our final results was ‘2A Outer Setting: Needs and resources of those served by the organisation’, usually interpreted as patients within a health care setting. We recoded this as ‘Needs and resources of students’ referring to those involved in the collaborative research projects, an aspect that emerged as important in project progression. A single researcher (RA) systematically coded transcripts to constructs outlined in the CFIR using the annotated codebook as a reference. Coded excerpts were reviewed by LH and KW, with discussion amongst the study team to refine understanding and interpretation of participant stories and arrive at consensus on the barriers and facilitators of key importance. Inclusion and exclusion criteria were refined iteratively throughout the analysis process, to manage overlap between constructs, ensure consistency and clarify concepts for the writing up phase. As familiarity with both data and use of the CFIR increased, the framework was modified slightly from the constructs identified a priori during interview guide development (Additional file 1). Where available, data from clinicians was triangulated with that from research fellow participants. Data organisation and coding was facilitated by use of NVivo (QSR International Pty Ltd). The lead researcher recorded changing ideas over the course of the study within a reflexive research journal, which alongside spreadsheet summaries of participant experiences and memos against CFIR template definitions, provided an audit trail of analysis.

## Results

Fourteen of 16 AH professionals approached consented to interview and recounted their experiences with student research projects. The majority of those working in clinical roles (10/11) were employed at senior or advanced practice levels. Two held post-graduate research qualifications (PhD, MPhil), two others were studying towards these and the remainder had either no prior research experience or limited involvement as team members on studies lead by other investigators. Clinicians described collaborations on 18 projects with 24 students, with three clinicians describing their experiences across multiple projects with different students. Research fellows described their formal or informal involvement in some of these collaborations, as well as involvement in other student projects additional to those described by clinician participants. Research fellow participants comprised three of five allied health research fellows employed within the health service at the time of the study and included two of the study authors (KW, LH). Participant demographics and student research project collaborations are summarised in Table 1.

**Table 1:** Description of participants and collaborations

Participants and collaborations	n
Total participants	14
Clinicians	11
Health service research fellows	3
Allied health discipline	6
Dietetics	6
Occupational Therapy	2
Pharmacy	2
Physiotherapy	2
Social Work	1
Speech Pathology	1
Age, mean years (range)	41.5 (29–55)
Female, n (%)	12 (86)
Male, n (%)	2 (14)
Universities	4
University departments	9
Dietetics	2
Occupational Therapy	3
Pharmacy	1
Physiotherapy	2
Social Work	1

**Fig. 1:**
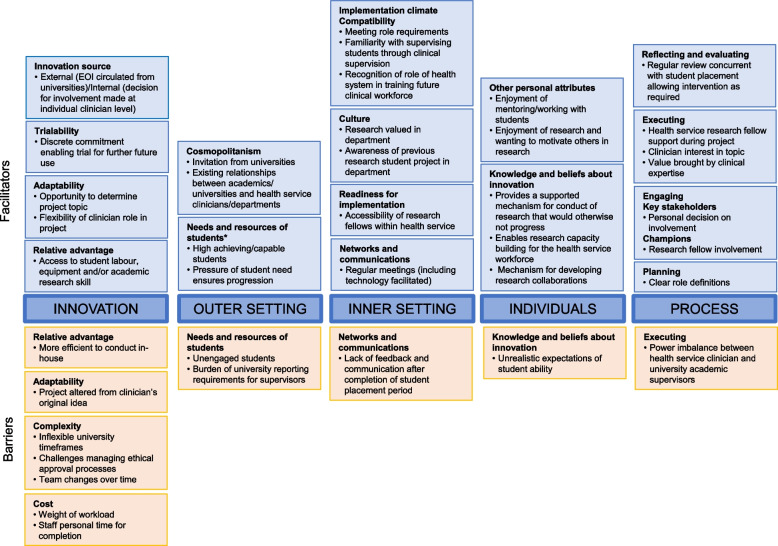
Facilitators and barriers to allied health collaborative student research projects mapped against the CFIR [11] *The construct “Needs and resources of students” was adapted from the original CFIR construct of “Needs and resources of those served by the organisation”, usually recognised as relating to patients, but which for the purposes of this study was recoded with reference to students

Use of the CFIR assisted identification of facilitators and barriers to both initiation of AH collaborative student research projects, and the smooth progression of these. These were mapped across all five domains of the CFIR, as summarised in Fig. 1. Twenty constructs (identified hereafter in *italics*) of key importance were identified, with several containing both positive and negative factors depending on the context of individual projects. Narrative description of the results is presented by CFIR domain. Exemplar quotes and other evidence for each category by CFIR domain are provided in Additional file 2. Additional file 3 provides a relative frequency estimation for the occurrence of the identified barriers and facilitators.

### Innovation characteristics

The combined internal/external nature of the *innovation source* facilitated project initiation. External to the health service, universities circulated expressions of interest requesting suggestions for collaborative student research projects, with the decision for involvement internally determined at the level of the individual AH professional based on their personal interest. The original research ideas were those of the clinician participants or were jointly developed between the clinician and university academic partner.

The student project collaborations provided a supported mechanism for conduct of research that would otherwise not progress, with *relative advantage* perceived in access to student time and labour, research skill of academic co-supervisors and/or equipment. These were major drivers for initiation of these collaborations which many participants also indicated were crucial to the eventual success of projects. However, there were also barrier aspects in this with some reflecting that in hindsight, conducting the study internally might have been more efficient considering the time invested by health service staff during student placements, or after to rectify problems with poor quality data collection.

*I’ve had two clinicians say, who have been through this say “I would never do this again. I would not have a student do this again. I can do this myself so much better. It’d be less cost, even though I’m a clinician, it’s less cost to the organisation for me to do the data collection properly.” P13 Research Fellow*

The *adaptability* of student research projects also had both facilitator and barrier aspects. Participants identified the opportunity to determine the research topic and flexibility in their role within the project as facilitators to involvement. However, this *adaptability* became a negative in a few cases where the focus diverged from the clinicians’ original idea over the course of the project. There was no mention of *complexity* in some participant’s description of their experiences. For others, barriers were created by the complex processes and timelines for obtaining ethical approvals for research, and in being bound to university timeframes. For example, one student placement co-coincided with the Christmas period where public holidays and high levels of hospital staff leave made completion of research tasks additionally challenging. One project had added complexity from research team changes over time which impacted its success. This arose from project extension to subsequent student cohorts and supervisory team changes to accommodate university and/or health service staff leave.

The discrete, time limited student placements provided an advantageous *trialability* aspect. After trialling collaborations as part of student projects, several participants extended research collaborations with their academic co-supervisors. In addition, one participant commented:

*It was almost like a good test to see whether we could utilise students a bit more in research, because there seems to be limited opportunities for clinicians to do research when they work full time. So, if this was going to be successful, then could we use this to get a few other projects [done]. P3 Clinician*

The most evident *cost* of the innovation was the weight of workload imposed upon the health service co-supervisors. When questioned, participants denied their involvement had impacted on their completion of clinical tasks. Instead, they reported use of their own time to ensure completion of both these and the additional responsibilities of student research project involvement, often recognising this would not have been sustainable over longer periods. Although a barrier to taking on students in the near future, these participants indicated they would seek involvement with student research projects at a later date, after they had had time to recuperate. Several indicated that the *relative advantage* provided by having students on these projects was sufficient to outweigh the *costs*.

*There’s always the time factor, that there is a little bit of time involved that, with meeting students and correcting, editing, there’s always time, but it’s a win, because they have done so much work that would have taken me way longer to do. P4 Clinician*

### Outer setting

*Cosmopolitanism* of the health service was a clear facilitator to the initiation of student project collaborations, evident in receipt of expressions of interest from nine different AH departments from four universities. The networking opportunities facilitated by health service-employed research fellows with conjoint or adjunct university appointments were also apparent from interviews both with research fellows, and with clinicians who had worked on projects with other research fellows outside of those participating in this study. A further facilitator was in existing relationships between university and hospital departments from student clinical placements, or between staff who had previously studied or worked at the other organisation.

The *needs and resources of students* involved in the collaborative projects had substantial impact on project progression. Several AH professionals described the responsibility they felt for students to achieve good project outcomes, and thus subject marks for their degree, and this sense of obligation facilitated the progression of research projects. Many referenced the competence, work ethic and capability of their student collaborators as critical facilitators in the success of the project. In other cases, student factors acted as a barrier, with challenges encountered with less capable or unengaged students. There was notable alignment between these student characteristics and whether the collaboration provided a facilitator or barrier aspect of *relative advantage* for project completion. An additional barrier in some cases was the work required by health service co-supervisors to meet university requirements, either in the administrative procedures for onboarding students, or in the review of student work for assessment purposes. In some cases, the latter constituted a substantial workload;

*I felt actually a little bit more overwhelmed with the project because I was more involved in providing feedback on drafts with the literature review… it’s such a big load providing – students coming in with no research experience and then all of a sudden they need to write this 15,000 word research report. P6 Research Fellow*

### Inner setting

The *implementation climate* was a clear facilitator of both initiation and progression of some research projects. There was tangible fit between student research project collaborations and health service values and work processes. This included *compatibility* with the expectation for research involvement for AH professionals employed at higher levels, as well as role requirements for supervision of students at all levels. Previous experience in supervising clinical placements gave first time research project co-supervisors confidence in initiating student research collaborations. Involvement was also compatible with recognition of the role of health services in training the future health system workforce. This was related to both clinical and research aspects of practice, respectively illustrated in the comments of these participants:

*When I realised the gaps in knowledge they have, I tried to… concentrate as much of that information and knowledge throughout our interactions, because I am convinced they will make use of it as a future professional. P1 Clinician*

*We’re building research capacity amongst a new generation of clinicians who are about to graduate, so they’ll be entering the workforce with some research experience. I know some of my previous honours students and some of my colleagues’ previous honour students now work at [our health service] and are doing research, which is excellent. P11 Research Fellow*

In some AH disciplines, aspects of departmental c*ulture* facilitated student project involvement. Participants described environments where research was valued and supported, and awareness of successful student research collaborations previously undertaken by colleagues.

The commitment of the health service to building research capacity provided a *readiness for implementation* facilitator. Research fellow support was critical to the progress of many student projects. In some, research fellows partnered with clinicians from the outset to provide research expertise and share the supervisory load. In others, they stepped in to support clinician supervisors where projects had stalled, or where relationships with university academics had broken down. Within individual projects, the importance of *networks and communication* were evident. Regular meetings were a key facilitator of success. Conversely, where communication was poor, projects often encountered difficulties. While in-person meetings between students and one or other supervisor were common, extensive use was made of communication technologies such as email and/or video conferencing, especially for between-supervisor communications. Indeed, one collaboration used email exclusively to reach publication stage without the academic and health service clinician supervisors ever directly seeing or speaking to one another, despite the co-location of their respective university and hospital offices within 500 m of one another. The importance of maintaining communications stretched beyond the student placement periods, enabling research publication and/or extension into further phases. In some cases, health service AH professionals never received copies of the final student results or theses, creating a barrier to pursuing further research in the area. Others indicated they would have valued feedback on their own performance to inform their future supervisory activities. Clinicians appreciated receiving invitations to attend students’ University-based final project presentations.

### Characteristics of individuals

The *knowledge and beliefs* of the participants involved in research student supervision influenced initiation and progression of projects in both positive and negative manners. Facilitators for initiation included the potential for longer-term benefits, such as developing collaborations with universities to support future work, and as a mechanism for building research capacity within the health service workforce in both future clinicians and current staff interested in learning research skills. Overlapping with the *relative advantage* construct, project initiation was facilitated by the belief the collaboration would enable research on a topic of interest which the health service employed AH professional otherwise lacked the time, research skill or equipment to complete. Often this belief was borne out in the successful result of the collaboration. However, in some cases, academic co-supervisors failed to provide the expected research expertise, or clinicians had unrealistic expectations of student ability which created barriers to project progression. For example, limited clinical knowledge and experience of students affected ability to interpret clinical notes and collect accurate data. As a result, requirements for extensive data checking and cleaning by health service AH professionals post-placement negated the anticipated benefits of student labour.

*I never supervised them going in, for example, doing a trial run of data collection and then coming back and going, “hey, is this clinically accurate?” which I think is really unfortunate because some of those errors were made – a lot of the errors were made in that first cohort. P2 Clinician*

*Other personal attributes* facilitating collaborative student projects included enjoyment of mentoring and working with students, and enthusiasm for research combined with a desire to motivate others to share this enthusiasm.

### Process

Additional aspects affecting initiation and progression of projects were coded in constructs described under the CFIR process domain. Level of *planning* varied considerably between projects. In a few cases, planning was aided by use of formal tools such as development and signing of memorandums of understanding, which may also have supported building of positive relationships. Role clarity for both research tasks and student supervision responsibilities facilitated both project progression and the enjoyment of the experience, as described by this participant:

*A good research team where everyone brings their expertise in, and then you just fit it together and everyone does their bit, and it’s just the communication, where you’re sitting around and everyone’s communicating well and everyone’s got their job, and then you start to achieve things. P10 Clinician*

A key facilitator in *engaging* was the opportunity for clinician co-supervisors (the key stakeholders) to determine the research topic, and individual-level decision for involvement in student research projects. The role of health service research fellows in acting as *champions* was also an evident facilitator of engagement, with several participants indicating these individuals had been a factor in their decision for involvement in a student research project.

*Execution* of projects was facilitated by the interest of the clinician supervisors in the specific topic area, along with their clinical expertise which facilitated data interpretation and application of clinical relevance to findings. Involvement of health service-employed research fellows, in either formal or informal roles, were key facilitators of success of many projects. These staff provided research expertise, shared student supervisory workload and in some cases, stepped in to assist with managing research relationships that had soured. In some projects, participants described power imbalances between clinician and university academics which had become a barrier to the progression of research. These participants described the value of support they had received from health service-employed research fellows, with some suggesting this should be an essential consideration for clinicians considering involvement in collaborative student research projects:

*I would then only ever do it again with a senior representative from our health service… someone to match the level of seniority [of the university academic co-supervisors]. P2 Clinician.*

No participants described a planned or formal process for *reflection and evaluation* during the conduct of their collaborative student research projects. However, when actively conducted during the placement period, these were clear facilitators. AH professionals related instances where concurrent evaluation enabled them to step in and take on new tasks to enable project progression, or to seek additional assistance (for example from health service research fellows) to resolve difficulties in project design or research relationships. In one case, where the student altered the study topic over the course of the placement, the participant described how reflection allowed her to move on from the project with minimal personal distress:

*I just realised at some point that I no longer was really a part of this project. P4 Clinician*

In projects where problems such as poor-quality data collection were later identified, evaluation and reflection had not been undertaken until after completion of the placement period.

## Discussion

We have previously identified health service benefits that can be obtained from staff involvement in collaborative student research projects [7]. Here, we used the CFIR to identify key facilitators and barriers to both initiation and progression of these projects. With improved healthcare performance associated with a research culture, the influences on clinician involvement in research have been much studied, and various frameworks developed to guide research capacity building in healthcare organisations [1, 14–16]. This study describes the facilitating aspects that collaborative student research projects can have for AH professional engagement in research and demonstrates the potential of these projects to overcome some previously identified barriers.

Insufficient time for research in the face of high clinical workloads is a common barrier faced by clinicians [17, 18]. Provision of funding to enable quarantined time for research has been indicated as an important supportive mechanism for clinician researchers [19]. However, it can be challenging to obtain competitive research funding and finding suitably qualified individuals to backfill specialist clinical roles is not always possible [20, 21]. Where students can take on more laborious tasks such as data collection, clinicians may use their limited time for higher level research tasks such as interpretation of results and application to clinical practice. This optimises the use of health service clinical expertise, while simultaneously supporting AH students to develop some basic research skills of value to their future clinical careers. A caveat is that clinicians must be aware of the need to provide appropriate training for student researchers in technical aspects of data collection. Otherwise, the benefits of student labour may not be realised.

Opportunities arising in the workplace can be central for a clinician research debut [18], and collaborative student research projects provide one such opportunity. However, not all clinicians want to become researchers [14, 20]. Staff who self-select as clinician-researchers may thus be the preferred targets for AH research capacity building strategies [14, 22]. The cosmopolitanism of our Health Service facilitated circulation of numerous student research opportunities, but these were only taken up by a subset of motivated clinicians. Efforts to expand staff involvement in student collaborative research projects must consider this. The individual decision to participate, combined with the opportunity to select the research area, were undoubtedly clear facilitators of project progression. Clinician engagement with research is greater when it is personally meaningful and helpful to them and the clinical population they work with [23]. Sense of ownership is an enabler of research [24] and here may have contributed to a feeling of responsibility for the project and student(s). Personal decision-making also likely selected for other facilitating characteristics such as enjoyment of mentoring, learning, and working with others, which may have helped offset identified barriers including the weight of additional workload that collaborative project involvement often imposed.

The value of research partnerships between researchers and knowledge users, including clinicians, is increasing recognised [15]. Partnerships can ensure that research is relevant to clinical needs, leading to greater uptake and translation into practice [3, 25]. They can also foster the development of research capacity in clinical workforces. From the health service perspective, partnering with academic researchers can enable clinicians to overcome the frequently reported barriers of lack of time, research skill, or equipment that is necessary for good quality clinical research [17, 18, 20, 22]. Indeed, all of these were aspects of the relative advantage of student projects that were identified by participants in this study.

Key principles identified by reviews of partnership research are evident in the findings of our study. One of the most frequently acknowledged is that partners build and maintain relationships based on trust, credibility, respect, dignity and transparency [26]. While for several participants, existence of previously established relationships with university academics was a facilitating factor for initiating collaborative projects, for others, the student project itself provided the opportunity to trial research partnerships that might potentially be carried forward into the future. The discrete time commitment enables both academic and clinicians to test the waters, and a means to build the trust and respectful relationships that are required for successful partnerships.

Any working relationship can have challenges, and partnership research is no exception. Issues may arise with power imbalances [24, 27, 28], as experienced by a few of our participants. Mutual respect and sharing power are recognised as key components in establishing productive research partnerships, and good communication is at the heart of any successful relationship [26, 29]. The development of respectful research relationships is supported by establishing ground rules which lay out clear goals for the study and roles for those involved, such that all parties know what each team member brings to the table [24, 27, 28]. As evident in the stories of some of our participants, establishment of a memorandum of understanding early in the partnership formation may aid this process. This does not preclude the adaptability that we identified as a facilitator for clinician involvement in student collaborative projects, and which has been previously recognised more generally as an important factor in partnership research [26]. However, our study indicates that reflection and evaluation during the course of the student placement is critical, enabling clinicians to recognise the need for change and optimise use of this flexibility. Communication lines must be maintained both during and after the student placement to share the research outcomes. Failure to do so, as observed here and elsewhere [23], may jeopardise future research partnerships.

Supportive and effective research relationships are important in building clinician research capacity, and the appointment of dedicated research staff within health services is a recognised facilitator of organisational research culture [16–18]. The value of health service research fellow support was evident in many of the participant experiences reported in this study, facilitating both project engagement and execution. As well as providing guidance in navigating research relationships with external partners, research fellows shared the student supervisory workload, and assisted in project design and conduct. Step by step guidance from a research fellow can alleviate the anxiety associated with fear of the unknown or failure in novice clinician-researchers [21]. Further, research fellows can provide advice to clinicians new to working with student researchers, helping them understand the training support required for completion of data collection tasks related to clinical activities, and thus short circuiting the problems that developed in a few studies where clinicians were caught off guard by the gap between their expectations and students’ actual capability. Clinicians require training to provide effective supervision to medical students on research placements [30], and a recent study found that novice supervisors were more likely to experience data acquisition problems than experienced supervisors [31]. Experienced supervisors were also more likely to favour a co-supervision model [31], perhaps in part because novice supervisors were unaware of the substantial commitments required for effective student supervision, combined with unrealistic expectations of student ability, as observed in our study. Not all student project collaborations will require health service research fellow support, however these may be particularly helpful for clinicians new to research, or for newly established collaborations. At the very least, clinicians engaging in collaborative student research projects should know where to seek assistance within their own organisation if issues arise.

### Strengths and limitations

The insider insight of the research team enhanced interview probing, ensuring better understanding of participants. We attempted to mitigate the intrinsic bias arising from our personal experiences with student projects through sharing interpretations of participant stories and robust discussion amongst the study team to reach a collective understanding. Use of the widely applied CFIR as a framework for analysis is a strength of the study that enabled identification of a comprehensive set of barriers and facilitators. However, the recognised complexity of the CFIR can make its use challenging for new users [32]. Discrepancies between the constructs identified a priori during interview guide development and those included in the final results are partially explained by increasing familiarity with the CFIR and its construct definitions that was developed during the analysis process. Consequently, not all determinants were explored with every participant, limiting the accuracy of frequency estimations for each. Our participants included representatives from a range of allied health disciplines but were employed within a single health service. Use of the CFIR will support comparisons with other settings. A recent update of the CFIR has seen its expansion to extend applicability across a range of innovations and settings, and improve alignment with other determinant frameworks [32]. While many constructs of importance identified in this study remain within the updated framework, some newer, potentially relevant determinants may have been overlooked. Advice mapping original constructs to the updated CFIR is available [32] which will facilitate use of our findings to guide other health services wishing to maximise the value of collaborative student research projects. Other resources available through the CFIR team, such as the CFIR-ERIC matching tool for selecting mitigation strategies for barriers to implementation may also be useful [33]. We were able identify both facilitators and barriers for project progression, alongside facilitators for project initiation. However, our purposive sampling strategy limited collection of information on barriers to project initiation, with participation restricted to those with experience in collaborative student research projects. We were thus unable to capture factors that may have prevented the involvement of otherwise interested clinicians.

## Conclusion

This study used the CFIR to identify the facilitators and barriers to the conduct of collaborative allied health student research projects. Health service cosmopolitanism, project adaptability and implementation climate (compatibility) were facilitators. Health service-employed research fellows provided readiness for implementation and a facilitator for project execution. Aspects related to project complexity and cost to staff in workload and personal time were the main barriers identified. The relative advantage of collaborative projects was affected either positively or negatively by differing student characteristics. Addressing these determinants when establishing each new project team may enable health services to optimise communication, role delineation and project success, and maximise the value brought by these projects, and thus ultimately, healthcare performance and patient care.

### Supplementary Information

**Additional file 1.** A priori versus final CFIR constructs: CFIR constructs identified a priori for investigation in comparison to those identified as of importance in final coding.

**Additional file 2.** Examples of supporting quotes mapped against the Consolidated Framework for Implementation Research (CFIR).

**Additional file 3.** Occurrence of facilitators and barriers to collaborative student research projects by CFIR domain.

## Data Availability

The datasets generated during this study are not available due to the sensitive and personal nature of the information contained. Data may be available upon justified request from the corresponding author with restrictions and following ethical approval.

## Abbreviations

CFIR: Consolidated Framework for Implementation Research
AH: Allied health
