# Supplementary material for: The health service perspective on determinants of success in allied health student research project collaborations: a qualitative study guided by the Consolidated Framework for Implementation Research
Source: BMC Health Serv Res. 2024 Jan 27;24:143. doi: 10.1186/s12913-024-10599-8 (PMC10821208; doi:10.1186/s12913-024-10599-8)
Supplement: Supplementary file 1 — Additional file 1. A priori versus final CFIR constructs: CFIR constructs identified a priori for investigation in comparison to those identified as of importance in final coding. [file 12913_2024_10599_MOESM1_ESM.docx]

Additional file 1: CFIR constructs identified a prior for investigation and those identified as of importance in final coding.

| **Identified *a priori*** | **Included in final results** | **Construct** | | **Short Description (CFIR template)** |
| --- | --- | --- | --- | --- |
| **I. INNOVATION CHARACTERISTICS**  *The act of health service-employed AH professionals supervising or co-supervising students on a clinically relevant research project undertaken as part of the students’ professional degree qualification program* | | | | |
| Y | Y | A | Innovation Source | Perception of key stakeholders about whether the intervention is externally or internally developed. |
| - | - | B | Evidence Strength & Quality | Stakeholders’ perceptions of the quality and validity of evidence supporting the belief that the intervention will have desired outcomes. |
| Y | Y | C | Relative Advantage | Stakeholders’ perception of the advantage of implementing the intervention versus an alternative solution. |
| - | Y | D | Adaptability | The degree to which an intervention can be adapted, tailored, refined, or reinvented to meet local needs. |
| - | Y | E | Trialability | The ability to test the intervention on a small scale in the organization, and to be able to reverse course (undo implementation) if warranted. |
| - | Y | F | Complexity | Perceived difficulty of implementation, reflected by duration, scope, radicalness, disruptiveness, centrality, and intricacy and number of steps required to implement. |
| - | - | G | Design Quality & Packaging | Perceived excellence in how the intervention is bundled, presented, and assembled. |
| - | Y | H | Cost | Costs of the intervention and costs associated with implementing the intervention including investment, supply, and opportunity costs. |
| **II. OUTER SETTING**  *The external social and political context including Australian and Queensland government policies and in particular, aspects related to universities in the local area offering allied health degree programs* | | | | |
| - | Y | A | Needs & Resources of students* | The extent to which student needs and their resources are accurately known and prioritized by the organization, including barriers and facilitators to student participation and contribution to the collaborative research projects. |
| - | Y | B | Cosmopolitanism | The degree to which an organization is networked with other external organizations. |
| - | - | C | Peer Pressure | Mimetic or competitive pressure to implement an intervention; typically because most or other key peer or competing organizations have already implemented or are in a bid for a competitive edge. |
| - | - | D | External Policy & Incentives | A broad construct that includes external strategies to spread interventions, including policy and regulations (governmental or other central entity), external mandates, recommendations and guidelines, pay-for-performance, collaboratives, and public or benchmark reporting. |
| **III. INNER SETTING**  *The employees, departments, systems, policies and resources of a tertiary hospital and health service located in south-east Queensland* | | | | |
| - | - | A | Structural Characteristics | The social architecture, age, maturity, and size of an organization. |
| Y | Y | B | Networks & Communications | The nature and quality of webs of social networks and the nature and quality of formal and informal communications within an organization. |
| Y | Y | C | Culture | Norms, values, and basic assumptions of a given organization. |
| Y | Y | D | Implementation Climate | The absorptive capacity for change, shared receptivity of involved individuals to an intervention, and the extent to which use of that intervention will be rewarded, supported, and expected within their organization. |
| - | - | D1 | Tension for Change | The degree to which stakeholders perceive the current situation as intolerable or needing change. |
| Y | Y | D2 | Compatibility | The degree of tangible fit between meaning and values attached to the intervention by involved individuals, how those align with individuals’ own norms, values, and perceived risks and needs, and how the intervention fits with existing workflows and systems. |
| Y | - | D3 | Relative Priority | Individuals’ shared perception of the importance of the implementation within the organization. |
| Y | - | D4 | Organizational Incentives & Rewards | Extrinsic incentives such as goal-sharing awards, performance reviews, promotions, and raises in salary, and less tangible incentives such as increased stature or respect |
| - | - | D5 | Goals and Feedback | The degree to which goals are clearly communicated, acted upon, and fed back to staff, and alignment of that feedback with goals. |
| - | - | D6 | Learning Climate | A climate in which: a) leaders express their own fallibility and need for team members’ assistance and input; b) team members feel that they are essential, valued, and knowledgeable partners in the change process; c) individuals feel psychologically safe to try new methods; and d) there is sufficient time and space for reflective thinking and evaluation. |
| Y | Y | E | Readiness for Implementation | Tangible and immediate indicators of organizational commitment to its decision to implement an intervention. |
| Y | - | E1 | Leadership Engagement | Commitment, involvement, and accountability of leaders and managers with the implementation. |
| Y | - | E2 | Access to Available Resources | The level of resources dedicated for implementation and on-going operations, including money, training, education, physical space, and time. |
| Y | - | E3 | Knowledge & Information | Ease of access to digestible information and knowledge about the intervention and how to incorporate it into work tasks. |
| **IV. CHARACTERISTICS OF INDIVIDUALS**  *Those of the people employed within the inner setting, specifically, AH professionals and specialist research staff* | | | | |
| Y | Y | A | Knowledge & Beliefs about the Intervention | Individuals’ attitudes toward and value placed on the intervention as well as familiarity with facts, truths, and principles related to the intervention. |
| Y | - | B | Self-efficacy | Individual belief in their own capabilities to execute courses of action to achieve implementation goals. |
| - | - | C | Individual Stage of Change | Characterization of the phase an individual is in, as he or she progresses toward skilled, enthusiastic, and sustained use of the intervention. |
| Y | - | D | Individual Identification with Organization | A broad construct related to how individuals perceive the organization, and their relationship and degree of commitment with that organization. |
| Y | Y | E | Other Personal Attributes | A broad construct to include other personal traits such as tolerance of ambiguity, intellectual ability, motivation, values, competence, capacity, and learning style. |
| **V. PROCESS**  *The process of conducting the collaborative student research project (the innovation) in its entirety from planning, engaging individuals within the inner and outer settings, executing the project and its evaluation* | | | | |
| - | Y | A | Planning | The degree to which a scheme or method of behaviour and tasks for implementing an intervention are developed in advance, and the quality of those schemes or methods. |
| Y | Y | B | Engaging (‘Key stakeholders’) | Attracting and involving appropriate individuals in the implementation and use of the intervention through a combined strategy of social marketing, education, role modelling, training, and other similar activities. |
| Y | - | B1 | Opinion Leaders | Individuals in an organization who have formal or informal influence on the attitudes and beliefs of their colleagues with respect to implementing the intervention. |
| Y | - | B2 | Formally Appointed Internal Implementation Leaders | Individuals from within the organization who have been formally appointed with responsibility for implementing an intervention as coordinator, project manager, team leader, or other similar role“ |
| Y | Y | B3 | Champions | Individuals who dedicate themselves to supporting, marketing, and ‘driving through’ an [implementation]” [101] (p. 182), overcoming indifference or resistance that the intervention may provoke in an organization. |
| Y | - | B4 | External Change Agents | Individuals who are affiliated with an outside entity who formally influence or facilitate intervention decisions in a desirable direction |
| - | Y | C | Executing | Carrying out or accomplishing the implementation according to plan. |
| - | Y | D | Reflecting & Evaluating | Quantitative and qualitative feedback about the progress and quality of implementation accompanied with regular personal and team debriefing about progress and experience. |

*Within the outer setting domain, *A. Needs & Resources of Those Served by the Organization* is generally recognised as relating to patients, but for the purposes of this study was recoded with reference to students.
