# Supplementary material for: The health service perspective on determinants of success in allied health student research project collaborations: a qualitative study guided by the Consolidated Framework for Implementation Research
Source: BMC Health Serv Res. 2024 Jan 27;24:143. doi: 10.1186/s12913-024-10599-8 (PMC10821208; doi:10.1186/s12913-024-10599-8)
Supplement: Supplementary file 2 — Additional file 2. Examples of supporting quotes mapped against the Consolidated Framework for Implementation Research (CFIR). [file 12913_2024_10599_MOESM2_ESM.docx]

Additional file 2 – Exemplar supporting quotes mapped against the Consolidated Framework for Implementation Research (CFIR)

| **CFIR domains and constructs** | **Type** | **Description** | **Exemplar quote or other evidence** |
| --- | --- | --- | --- |
| 1. **Innovation Characteristics**   The act of health service-employed allied health professionals supervising or co-supervising students on a clinically relevant research project undertaken as part of the students’ professional degree qualification program | | | |
| A. Innovation source (idea for research project and decision to co-supervise a student) | | | |
|  | Facilitator | External (EOI circulated from universities)/Internal (decision for involvement made at individual clinician level)  Idea for research project | Involvement in the supervision of collaborative student research projects was undertaken by interested health service AH professionals on an *ad hoc* basis, in response to (external) University calls for expressions of interest. The decision for involvement is made at the level of the individual clinician.  *My colleague and I came up with a concept after Journal Club, and a bit of earlier lit[erature] searching... We eventually pitched to [University] the following year. P2 Clinician*  *The idea was mine. P4 Clinician* |
| C. Relative Advantage | | | |
|  | Facilitator | Access to student labour, equipment and/or academic research skill | The other option would have been me doing it, which was at that point not feasible, just because of time constraints. P7 Clinician  Unless we had the student it wasn’t going to happen. Yeah, there just isn’t capacity for the clinicians to do research… unless we have students we’re not really going to get anything done. P6 Research Fellow  I noticed that there’s issues or there’s problems that could benefit from a bit of focus and research, but I never had, I guess, the job or the understanding of the research process to drive this further. That’s when these guys came in the picture and starting very quickly – they very quickly identified it as a research opportunity and pitched some ideas on how we could address this. Because they had a student available, we just thought we could use this and involve a student as part of the Master’s project, graduation project, and yeah, make it happen that way P1 Clinician  I’m in a senior clinical position and there’s probably some expectation with having a PhD that you continue to do research. So then this was a way that I can – if someone said, what research have you been participating in, it was something that I could put forward and say, we’ve been looking at this and doing this without spending all my clinical time on it. P8 Clinician  [University] had the research expertise and were helpful with the research question that we wanted to answer. Without their equipment – because the testing equipment was, I think over $35,000, so for us to buy that, it just would never have happened... The student time was imperative to get that done. P14 Clinician |
|  | Barrier | More efficient to conduct in-house | It did seem like it was a lot more trouble than it was worth in the end. So they did complete the data collection that we'd asked, but it wasn't very accurate, so at the data cleaning stage it took me a lot of time to go through and basically review everything that they had done. So I don't think that it really made our life much easier. I think it ended up taking just as long, if not longer, if I would have done it myself. P3 Clinician  With the data and the outcomes that we’ve got from the study and considering the time and resources that were invested in it, yeah, I think there’s a bit too much time invested for what we’ve got out of it. P11 Research Fellow  I've had two clinicians say, who have been through this say “I would never do this again. I would not have a student do this again. I can do this myself so much better. It'd be less cost, even though I'm a clinician, it's less cost to the organisation for me to do the data collection properly.” P13 Research Fellow |
| D. Adaptability | | | |
|  | Facilitator | Opportunity to determine topic  *(research question and study design devised by Health Service staff, or in collaboration with University academics)*  Flexibility of clinician role in project | The reason why I picked that was solely because I was already working in that space. If she had said she wanted to do a systematic review in something else, I probably wouldn't have taken her on as a student. P9 Clinician  We chose to do a systematic review on a topic that I presented to them. This was something that I was interested in, a gap in the research that we thought would be a publishable study. P8 Clinician  It was just based on my discussion with some of the [University] staff, and I guess through previous involvement with some of that staff with other students... We just started discussing about the gaps and knowledge with regards to [research topic] and I can’t remember exactly who spun the idea. But basically, we all agreed that it’s a good idea and we should try and see what exactly is the situation. P1 Clinician  I could contribute as much or as little… at the beginning they had conversations about well, if you contributed a certain amount, then you’ll get an authorship and otherwise, you won’t. So, I was mindful of that and motivated. P5 Clinician |
|  | Barrier | Project altered from clinician’s original idea | I actually had a completely different project idea… I’m interested in research, and I like being involved in it, but I don’t do it for its own sake. So, I go into it for a reason… there’s a gap in practice, or there’s a question you want to be answered, and that just got completely flipped into something totally different. P10 Clinician  The literature review that was done was completely not what I wanted or what was helpful. P4 Clinician |
| E. Trialability | | | |
|  | Facilitator | Discrete commitment enabling trial for further future use | It was almost like a good test to see whether we could utilise students a bit more in research, because there seems to be limited opportunities for clinicians to do research when they work full time. So, if this was going to be successful, then could we use this to get a few other projects [done]. P3 Clinician |
| F. Complexity | | | |
|  | Barrier | Inflexible university timeframes  Challenges managing ethical approval processes  Team changes over time | People, you know were away and things like that – I was away. So it wasn’t the best of time, but it was necessary to student’s schedule… they are bound to a certain timeline. P1 Clinician  Around about September was extremely stressed because of the whole timeframe issue. I knew that it just wasn’t ideal time for us to do surveys over December. P6 Research Fellow  Not knowing all the different processes and paperwork that's required to actually have students on board… Some of the barriers perhaps is just around the processes involved and how long things take. I know we had quite a big challenge waiting for the [ethical approvals] for the student to finally start their placements. P7 Clinician  There was some delays with the ethics, and that was the biggest problem. P1 Clinician  One issue was that the clinical staff involved in the project and the university staff – or the university supervisors, across the board there were lots of changes in staffing. I know that people went on maternity leave, changed jobs, moved overseas, there was lots of movement… There was no backup [within the Health Service co-supervisors] so that if this person went on leave this is the person who is now the lead, and the same with the university academics. P11 Research Fellow |
| H. Cost | | | |
|  | Barrier | Weight of workload  Staff personal time for completion | In my position, whatever I don’t do now I’ll have to do later, I can’t delegate it… you can select the jobs that can be delayed. But I did feel an increase in the workload, and I felt increased pressure because of this definitely. P1 Clinician  There was more pressure to fit in my clinical work during that six weeks. I made it work but it was a very busy six weeks. It wouldn’t be sustainable ongoing like that. P8 Clinician  *it is a big-time investment, but I think that was expected from the beginning. P7* *Clinician*  I didn’t really have time set aside for that so that just had to be squeezed in. P8 Clinician  I did do – probably about 50 per cent of the work is in my own time, yeah – or more I would say. P1 Clinician  It did take a fair bit of my own time when I was on parental leave. So I'd spent a fair bit of time reading articles and responding and things like that and that was sometimes hard. P5 Clinician  *It flowed on into my maternity leave and so I had a newborn whilst writing up the paper with the student… The negative is that personal time was put into it. P8* *Clinician* |
| **II. Outer Setting**  The external social and political context including Australian and Queensland government policies and in particular, aspects related to universities in the local area offering allied health degree programs | | | |
| B. Cosmopolitanism | | | |
|  | Facilitator | Invitation from universities  Existing relationships between academics/universities and health service clinicians/departments | Participants described projects initiated in response to expressions of interest to collaborate on student research projects extended to the health service from nine different university allied health departments (five allied health disciplines from four universities)  There was an email from [University] at some point to say that they were looking for student projects for their next intake of students. P12 Clinician  There was an opportunity; every year they always sing out for projects. P5 Clinician  The first academic was, I think, actually a clinician here previously. Then the second academic was [in a research role] here at one point. P13 Research Fellow  They were from [University] and that's where I did my research from, so I do have a few links with the unis at the moment. P3 Clinician  We were really excited… and we were all very early in the Clin Ed (clinical education) relationship with [University] as well and it was all very positive. P2 Clinician  [University Academic], who I’ve known for many years and often do a few research projects with, he gave me a call one day and mentioned he had some new equipment... He asked would I be interested in brain-storming up a project with one of the Honours students, and I said, yep, great. So, I met with him several times leading up to the Honours student commencing, and we devised a bit of a – some methods of what we were going to look at. P14 Clinician |
| B. Needs and resources of students* | | | |
|  | Facilitator | High achieving/capable students  Pressure of student need ensures progression | Working with the student was a good experience – he was a particularly good student. I think that was a big part of it; I think it’s absolutely essential to have a particularly good student. P1 Clinician  *One of the students was great and really enjoyed it, so that was a good experience. P3 Clinician*  It was such a great bunch of students that they were really responsive and so respectful, and produced a very high level of work, and, when I did give them some feedback, they adjusted things with no problems at all. P4 Clinician  I had one student who was – who just sat back and went for the ride and then another student who did a lot of work and she was amazing… The easy bit was me saying what to go and do next and they go and do it and it gets done. Like that was amazing and I feel like that was very student dependent. P8 Clinician  *The student that we got was really good, in fact he went onto study medicine – he was very good. P10 Clinician*  *I’m always quite proud of my students because by the end of the project they’ve learnt a lot and they conduct themselves well. P11 Research Fellow*  *She was passionate about the area. She was a hard worker. P12* *Clinician*  *This student was really good and industrious P13 Research Fellow*  She was quite astute and she was capable, I sort of let her drive it. But if you had a student who wasn’t as forthcoming and – it might be different. P14 Clinician  The expectation and time pressure which partly is for myself - it’s not like someone put that on me. But when you commit to something and start something it’s hard to pull out halfway through when you’ve got other people involved. P8 Clinician  *I was worried that the student wouldn’t have any results. That was the big worry. P6 Research Fellow* |
|  | Barrier | Disengaged students  Burden of university reporting requirements for supervisors | We had four students and there were definitely varying levels of engagement. So you had ones that were quite interested and continued to ask questions and wanted feedback, but then there was a couple of others that probably weren't really interested in research and they probably just did this because the uni made them do it. P3 Clinician  *Some of the students' attitudes. You could clearly see that they weren't enjoying it or didn't really want to be here and they were just doing it to satisfy [course requirements]. P3 Clinician*  *I had one student who was – who just sat back and went for the ride and then another student who did a lot of work and she was amazing… The easy bit was me saying what to go and do next and they go and do it and it gets done. Like that was amazing and I feel like that was very student dependent. P8* *Clinician*  *Facilitator: Was that a personality trait of the student?* *Interviewee: I think there was that element of a personality trait. So that's why I think that one was difficult. P13 Research Fellow*  *The students just don't even have that level and they're not being trained to. They haven't even got clinical experience. So if it's just, you can pull some data and they can look at it and write something up, then write up a paper or whatever. P13 Research Fellow*  I felt actually a little bit more overwhelmed with the project because I was more involved in providing feedback on drafts with the literature review… it’s such a big load providing – students coming in with no research experience and then all of a sudden they need to write this 15,000 word research report. P6 Research Fellow  I was quite conscious that I actually had to meet her learning goals, as a student, as opposed to what the research goals of delivering a publication in a particular space. So, it wasn't about, let's just get to the end goal and that's what we need to do. We had to tick a lot of boxes to say that you were could actually walk away and do this again, on your own. So some things took a lot more time. Some things we did several times, when, in research, we would have just done it once and it's done - leave it. Things like developing a protocol and an ethics - we did that process a few times, so she knew what that looked like, and did it on her own, and then I did it as well. So everything took longer. P9 Clinician  It can be quite stressful, a lot of pressure. Especially for the supervisor reading drafts of the thesis. P11 Research Fellow |
| **III. Inner Setting** The employees, departments, systems, policies and resources of a tertiary hospital and health service located in south-east Queensland | | | |
| B. Networks & Communications | | | |
|  | Facilitator | Regular meetings (including technology facilitated) | The guys from [University] set up a very good Teams-based communication, you know forum, and that worked really well. So they were quite diligent in sending appointments and keeping the appointments and things like that. So that worked really well. P1 Clinician  Mainly face-to-face meetings, lots of emails I guess in the beginning. But then probably mainly face-to-face meetings to nut out the big things and then emails to follow-on. Fairly frequent at the beginning to nail it all down and nut it out and plan it, and then it eased off, and then every time you’d have a new step that you had to work out, then we’d probably have another meeting. P10 Clinician  We did video calls leading up to the six weeks and then in that six weeks we had a couple of sessions here together and that was without the Uni educator. So they [the students] would come here, we’d have a session, and then they’d go off and do some work and then we’d have email correspondence and phone conversations in that six weeks quite a bit and then mainly email after… We continued with email correspondence and phone calls after the placement to get [the publication] done. P8 Clinician  *We had weekly meetings. P6 Research Fellow*  *That was easy. We didn't do anything outside of her placement hours. Everything was done one-to-one, in person. P9* *Clinician*  *In terms of location, the university's quite close. So, we met face-to-face with all the team a couple of times and then a lot obviously with email. P12* *Clinician*  Antithesis – Problems arising from insufficient meetings and communication planning:  *That one struggled in terms of adequate communication… there was no communication plan because there was no MOU [memorandum of understanding]. Then yeah, I think between even the student cohorts there maybe wasn’t good handover or communication there as well, so yeah, lots of communication problems. P11 Research Fellow* |
|  | Barrier | Lack of feedback and communication after completion of student placement period | If I am to do this again, it would be good to have some feedback – I’m happy to get negative, positive, whatever... there’s a lot of thank yous and that sort of formal, but maybe a bit more targeted feedback from the student in particular would be good. P1 Clinician  There was a survey conducted and it was written up, but I never got a copy of the final, despite having asked for it a few times. P4 Clinician  Facilitator: Have you received a copy of the thesis? Interviewee: No. I’d like – I’d love to read it [laughs]… From the little bit of information I’ve been provided with at the oral presentation day, yes, but I’d like a bit more. That’s why I’d like to see the report, so I can try and act on some of the stuff that was picked up. P14 Clinician  *When that student finished, there was a report that was sent to the doctor in that area, rather than us. We had to actually go and [get it from them]. P13 Research Fellow*  *Facilitator: Did you ever see the final report? Interviewee: No. Facilitator: Did you have any opportunity to go and see any presentations on that from the students, even if you did or didn't go? Interviewee: Wasn't invited, don't know if it actually happened. That was the intention, that they would present something back. Facilitator: That's something you would have liked to have done? Interviewee: Oh yeah. It would have been interesting just to see what data – or what they analysed from the data that we had from our patients. So yeah, no, it would have been beneficial to see them. P3* *Clinician*  Antithesis – Appreciation of University invitation to student research presentations:  …an invitation to come and watch them present this at [University]. Which I then did. And they thanked me very much for my contribution… I really enjoyed it. It was a really nice thing to see - I felt like my idea displayed for the world to see. They’ve done such a good job, and they had understood the intent of what I wanted them to do. P4 Clinician |
| C. Culture | | | |
|  | Facilitator | Research valued in department  Awareness of previous research student projects in department | This study exhaustively recruited eligible participants across all allied health disciplines within the Health Service. The higher number of participants (5/11 clinicians) from the only allied health department discipline in which a dedicated research fellow was employed is an indication of a cultural facilitator.  It’s helpful having someone like [Research Dietitian] as well that’s a part of your actual department, who’s in your profession, and part of the department, who can help facilitate the process as well. P10 Clinician  As long as I get my clinical work done and it doesn’t impact it then I’m allowed to help, then do research. P8 Clinician  There has been other areas of [allied health discipline] where they've utilised students to do research projects, I guess similar sort of things, some doing data collection, some doing literature reviews. They'd had good success with it, so we felt, yeah, why not give this a shot? P3 Clinician  So we, I think, ran a couple of student projects with [University] in the past and from those other projects they seem to be able to bring about some reasonable results. P12 Clinician  I’d heard that other people had had [University] students and that it could be helpful to undertake research with them and that it’s sort of a give/take type relationship that they get a topic and experience or access to a database and you get help to complete a research project. P8 Clinician |
| D. Implementation climate | | | |
| D2. Compatibility | | | |
|  | Facilitator | Meeting role requirements (for research and/or student supervision)  Familiarity with supervising students through clinical supervision  Recognition of role of health system in training future clinical workforce | It was also to supervise the students who were research dissertation and support a clinician in that process. So that it’s really about capacity building… it’s part of our role to build research capacity. P6 Research Fellow  Every six months, we get asked if we'd like to take a student within the [discipline] department, so if we're out for two years we are supposed to take on a student. P9 Clinician  I’m in a senior clinical position and there’s probably some expectation with having a PhD that you continue to do research. So then this was a way that I can – if someone said, what research have you been participating in, it was something that I could put forward and say, we’ve been looking at this and doing this without spending all my clinical time on it. P8 Clinician  I’ve had many other students, but not to do with research. P4 Clinician  I had students before, for smaller projects, qualities of medicines and the like. I knew some of the students that I had in the past, I knew some of them are quite good and quite motivated. P1 Clinician  To influence their [Universities] program to make sure that their students were, I guess, having some exposure to some of the real-life of working in a hospital, or health service and those kinds of dynamics as well. P5 Clinician  I really think it’s great exposure for students to see how clinics work. Especially the area that I work in, because it’s quite a specialised area. So, the more people I can get through my clinics, the better it is for the profession and for those individuals. P4 Clinician  My focus has been though, to a large extent, educating the student. When I realised the gaps in knowledge they have, I tried to… concentrate as much of that information and knowledge throughout our interactions, because I am convinced they will make use of it as a future professional. P1 Clinician  We’re building research capacity amongst a new generation of clinicians who are about to graduate, so they’ll be entering the workforce with some research experience. Which I know some of my previous honour students and some of my colleagues’ previous honour students now work at [our health service] and are doing research, which is excellent. P11 Research Fellow |
| E. Readiness for Implementation | | | |
|  | Facilitator | Accessibility of research fellows within health service | Support was mainly from [Health Service Research Fellow], so in terms of just getting the students, so that help and that connection with the unis, applying for ethics and the processes involved with that, so just working collaboratively with that. P7 Clinician  If I didn't have [Health Service Research Fellow] sitting in that space, all of these questions would have been different in terms of the challenges, because I would have been navigating, literally, in the dark, of what I'm supposed to be doing. So, yeah, without that academic guidance, I don't think it would have been as successful as what it was, and I don't think I would have put my hand up to take a student… I wouldn't have done it without that academic expertise and support. It wouldn't have happened. P9 Clinician  I required a lot of sort of I guess support along the way from [Health Service Research Fellow] about how to handle some of these more ethical issues of two very powerful, well-known international research - respected researchers saying... P5 Clinician  I was very surprised at the poor communication processes. The fact that there were no roles. I was unhappily - I was disappointed to see the lack of communication, particularly on that systematic review, and I was frankly confused, because I thought, what are [ the Academic co-supervisors] doing? When I'd asked the clinician all these questions, she goes, well I don't know, they've not said anything. After about five, ‘I don't knows’, I thought, what's happening here? What what's going on? That's why I said, right, I need you to sit down, I want you to write an email to all of them requesting a meeting, and we're going to start putting, and we're going to have minutes from every meeting. We're going to start putting down goals and timeframes and who's doing what. P13 Research Fellow  *Interviewer: What about ethics – did you get any help with that? Participant: My colleague helped with that. Interviewer: Within [the Health Service]? Participant: Yes. P1* *Clinician* |
| **IV. Characteristics of Individuals**  Those of the people employed within the inner setting, specifically, AH professionals and specialist research staff | | | |
| A. Knowledge & Beliefs about the Innovation | | | |
|  | Facilitator | Provides a supported mechanism for conduct of research that would otherwise not progress  Enables research capacity building for the health service workforce  Mechanism for developing research collaborations | It was actually quite a relief knowing that we could get students doing some projects for us and doing what was probably going to be quite a labour-intensive process and knowing that this project could then actually progress with the help of students. P7 Clinician  We had a research project that had already started. It was a multicentre study just doing a lot of respective data audits, data collection and analysis. Being a full-time clinician, it was hard to get the time to do it. So, the opportunity came up to take some students and we thought that'd be a good time to have four students with five weeks of just doing the data stuff for us. P3 Clinician  I noticed that there’s issues or there’s problems that could benefit from a bit of focus and research, but I never had, I guess, the job or the understanding of the research process to drive this further. That’s when these guys came in the picture and starting very quickly – they very quickly identified it as a research opportunity and pitched some ideas on how we could address this. Because they had a student available, we just thought we could use this and involve a student as part of the Master’s project, graduation project, and yeah, make it happen that way P1 Clinician  Not having done any research before, I felt quite overwhelmed in starting a research project on my own. So I was hoping to learn some of the skills and the process involved in research without having to do, I suppose, 100 per cent of the groundwork. P12 Clinician  It’s good for [our health service] to be supporting research capacity building, even in students. Because yeah, they might become part of our workforce one day. P11 Research Fellow  *We’re building research capacity amongst a new generation of clinicians who are about to graduate, so they’ll be entering the workforce with some research experience. Which I know some of my previous honour students and some of my colleagues’ previous honour students now work at [our health service] and are doing research, which is excellent. P11 Research Fellow*  *The plan is now – next, is to go through the same process, is to build research capacity for –through them being involved in this next student, yeah, hopefully they get exposure to the research cycle. P6 Research Fellow*  *We had no research experience so given that we had two lecturers at the time, one with a significant amount of research experience and another not, we had hopes for more than just an observational study in the longer term. We thought that this could become an ongoing project that we would continue to I guess build our own research capacity in. P2* *Clinician*  *Actually learning different things, so they did have the support of the statistician at [Name of] University, because there were some fancy stats, I call it, that was required, so just getting that knowledge and being able to have that resource as well I think was really good. There's definitely things that I can personally then use in future projects as well. P7* *Clinician*  Having those collaborations with the unis, I think that's probably a good thing. P7 Clinician  I think it's good to make those links with the University just in terms of being a researcher... it's always good to get them involved. P3 Clinician  *Even though we’ve got [discipline academics] in the school over at [University] doing research and we’ve got [discipline clinicians] here at the hospital doing research, they’re not necessarily well-connected in terms of building research collaboration. I think these student projects are a nice little opportunity to start connecting some of the university researchers with the hospital clinicians. P11 Research Fellow*  I liked the - that sort of building some connections and relationships with the university and the students. P12 Clinician  *I enjoyed working with [University] because there’s just so much we can do with the university, because basically they’re 100 metres away. They have a massive bucket of students which we can utilise to get some good data and research happening, which is going to benefit them and us together. I think it’s a shame that we don’t use that partnership more at times, but hopefully this is – we can start doing that. P14* *Clinician* |
|  | Barrier | Unrealistic expectations of student ability | One issue was that on a longer project there was I think three different cohorts of students collecting data on that study and they weren’t all trained in the same way. Then the data we got was pretty inaccurate and we had to get rid of some of that data. P11 Research Fellow  The students just don't even have that level and they're not being trained to. They haven't even got clinical experience. So if it's just, you can pull some data and they can look at it and write something up, then write up a paper. P13 Research Fellow  I never supervised them going in, for example, doing a trial run of data collection and then coming back and going, hey, is this clinically accurate? Which I think is really unfortunate because some of those errors were made – a lot of the errors were made in that first cohort. P2 Clinician  *I think we were expecting too much of the students or I suppose we didn’t know what their level of understanding would be. Because we assumed that having done their clinical placement, they are technically qualified to practise. Yeah, so the areas that we were seeing in their data collection for the clinical skills in the research activity we were sort of floored that it was that poor. P2 Clinician*  What I thought was a simple task to give them was probably a little bit beyond what I thought they were capable of. I think a lot of it does relate back to the clinical knowledge that they were lacking to be able to realise that, this is not accurate, what I'm typing, the patient can't have this much of this or this much. So I think there was – noticed the big gap between clinical knowledge and experience to be able to just interpret data. P3 Clinician  You need to be aware that students don't have the same level of understanding or clinical background as a clinician doing research. Even the most basic things are sometimes beyond the scope of a student, so - need to be very selective about the task that I would give a student to do if they're involved in a research project. They potentially need a lot more direct supervision. P3 Clinician  *For the student to learn or to accumulate the knowledge and then translate that into something meaningful will just take too much time – I was not realistic P1* *Clinician* |
| E. Other Personal Attributes | | | |
|  | Facilitator | Enjoyment of mentoring/working with students  Enjoyment of research and wanting to motivate others in research | I really enjoyed that aspect, but that’s part of what my favourite part of my job is, is support - you know, developing - seeing people transition and develop and that kind of thing. P5 Clinician  I'm always really passionate about looking after what they actually need and getting a sense from what they need. So sometimes, they need that emotional well-being stuff, sometimes they need process stuff and sometimes they need to be stretched in their clinical reasoning. So whatever it was, I would do my best to contribute to that or provide that. P5 Clinician  I don’t mind educating. I like that sharing of skill and information. P8 Clinician  Being able to impart some knowledge on to some people as well. P7 Clinician  One of the things that's important is to make sure the students have a good learning experience as well, so really just having that at the back of my mind to say, okay, we need to give students a good learning experience, I think, yeah, it's always at the back of my mind. Hopefully that was something that they were able to get. P7 Clinician  I really, really enjoy having students, and I like sharing my ideas and sharing my knowledge. P4 Clinician  I liked the - that sort of building some connections and relationships with the university and the students and I suppose even just from a supervisor point of view is watching the student learn those skills and develop and all of that as well. P12 Clinician  *I wish I could have made it more of a priority – spend more time on it, spend more time with the student…Working with the student was a good experience P1* *Clinician*  Evidenced by two clinician supervisor participants going on to enrol in PhDs, and others already being enrolled or having a higher degree by research (HDR).  I enjoy research and I enjoy that other people – or that you can potentially influence other people to enjoy research. P3 Clinician  I’m passionate about [clinical area] research. P6 Research Fellow  I just don’t want it to stop now that the Honours has been done and dusted, I want to keep on going. P14 Clinician  Just hoping that the students have a good experience in their research projects. So that was the major things, that they got a good experience and hopefully motivate somebody to actually then continue with research. P7 Clinician |
| **V. Process**  The process of conducting the collaborative student research project (the innovation) in its entirety from planning, engaging individuals within the inner and outer settings, executing the project and its evaluation | | | |
| A. Planning | | | |
|  | Facilitator | Clear role definitions | It was really well outlined from the start, and we rolled it all out. Whenever I’ve worked with these teams in any research, it’s always outlined from the beginning, and documented and agreed upon, so it’s always clear, so that’s good. P10 Clinician  *A good research team where everyone brings their expertise in, and then you just fit it together and everyone does their bit, and it’s just the communication, where you’re sitting around and everyone’s communicating well and everyone’s got their job, and then you start to achieve things. P10 Clinician*  *We sort of had things outlined in terms of the expectations and the requirements, I suppose, as a supervisor… it was always clear that we were going to have quite intensive involvement with the students. So I don't think it differed very much from what I would have expected.* *Facilitator: Was there any written agreement or MOU in place?* *Interviewee: Yes, so there were some agreements… it was clear who was responsible for the students and what their role was in supporting the student and with the data collection, where they were going to get the data. It was also clear in terms of – because we're working as part of an MDT, who was going to collect another set of data or the names and how that would then be translated for the students do then do the extra work. … I think it was probably clear in terms of who is doing what in terms of that and the supervision. P7 Clinician*  *Interviewer: How about then a clear – did you have a clear expectation of what your involvement would be and what their involvement would be?* *Participant: Yeah, we did talk about that at the beginning, and I did state very clearly about – that I wouldn’t be able to do the clinical part and that my timeframes with maternity leave and what I could offer from a time point of view which did pretty much eventuate, plus a bit extra. P8* *Clinician*  *Was that clearly outlined at the start of what your role would be and perhaps what the academic's role would be in supervision?* *Interviewee: Yeah, yes.* *Facilitator: Did that go as you expected?* *Interviewee: Yes. P12* *Clinician* |
| B. Engaging | | | |
| B. Engaging | | | |
|  | Facilitator | C1 Key Stakeholders: Personal decision on involvement (cross-coding with Innovation source)  Champions (Research fellow involvement) | Involvement in the supervision of collaborative student research projects was undertaken by interested health service allied health professionals on an ad hoc basis, in response to University calls for expressions of interest.  *I’ve said to [University academic] over the years, we need to try and get more clinical work relationships happening with the hospital, because there’s a lot of public health stuff going on, but it’s always been difficult to establish that partnership here. But now I think, with all these research positions that we have here, it might help facilitate that partnership. P14 Clinician*  *Having the research dietitian there, being able to connect to different universities. P7* *Clinician*  *My direct line manager was [a high-level research position within the health service] who is obviously very keen on research and pushing connections with unis and thought it was a really good idea. She thought with her qualitative background that she'd help to support it as well. So, we put in an initial application together. P5 Clinician*  *We let the clinician pick the topic and then we said, okay, so it’s a mental health topic. I’ll provide support to him. So, I was the associate or co-supervisor and specifically provided support with the ethics application, all of that. P6 Research Fellow* |
| C. Executing | | | |
|  | Facilitator | Key stakeholder (clinician) interest in topic  Value brought by clinical expertise  Health Service Research Fellow support | Responses to being questioned if they would recommend co-supervision of a collaborative student project to other clinicians:  If they can do a research project that’s going to benefit their practice and help them answer a question that potentially is in the back of their mind, then definitely, yeah. P14 Clinician  *Make sure you pick something interesting. I think that made a big difference for me, because it was something that I found quite exciting and interesting. You will need motivation because when you have competing jobs and competing duties, if you don’t have something exciting, it’s very, very easy to get side-tracked. P1 Clinician*  You need to understand like the clinical management of the ward to give it context. P10 Clinician  I probably provided the clinical perspective for the application of the tool and how it would practically be applied or whether it was useful like in a clinical context, like day-by-day management of the ward. I guess I provided like education to the student on the use of [electronic medical record]. P10 Clinician  It was important to offer the student an overview of the health system, how the health system works, so they can integrate that information into the design of the survey. P1 Clinician  *One big area that I think a lot of clinicians can provide a lot of insight and really help the research team… is in data analysis and interpretation and writing up… they want to hear what the findings are and then I’ll want to discuss it with them in the context of the clinical area or clinical practice. Then obviously in terms of writing that up we’ll have their spin or their insights to add. P11 Research Fellow*  *I think that we probably have a lot more to offer than perhaps it’s considered from external bodies. I think that was a shame that clinicians might be underestimated. I think they actually have a wealth of specific knowledge that is not in existence externally. P2 Clinician*  *It was important for the student as well to understand the processes and things around our clinical treatment and management for this population and that there are some guidelines and clinical recommendations made around treatment and therapy. I think it was important for her to understand that background. P12 Clinician*  I would then only ever do it again with a senior representative from our health service… someone to match the level of seniority [of the university academic co-supervisor] P2 Clinician  I needed [Research Fellow] in the room in order to get my point across sometimes, you know? So they were using their power differentials a bit too. P5 Clinician  If I didn't have [Research Fellow] sitting in that space, all of these questions would have been different in terms of the challenges, because I would have been navigating, literally, in the dark, of what I'm supposed to be doing. So, yeah, without that academic guidance, I don't think it would have been as successful as what it was, and I don't think I would have put my hand up to take a student, having just learned more about research, because I'd been a participant in the past. There is no way I would have felt confident to take a student…. I wouldn't have done it without that academic expertise and support. It wouldn't have happened. P9 Clinician  *without that there’s no way that I would have been able to do this at all. Because yeah, clinicians, certainly any research that I’ve done is basically the clinical role and then the collecting of the data around that clinical management, and contributing that, rather than the research side. So yeah, so from the professor [employed within the Health Service] that was involved, so essentially her guiding the research process, and just all the stuff that’s involved in that, like the documentation and the flow processes that have to happen…I wouldn’t have known any of that... So basically that each step of the research process, basically got help from that, and from [Health Service Research Fellow] as well – her input from that point of view. P10 Clinician*  *[Research Fellow] is experienced and she knew what she wanted with the papers we were writing, but if you were a novice researcher, then it could have been a little bit tricky. If I was to supervise an Honours student without any help from [University], I might be in the deep end a little bit, because I – even though I’ve been a team member on several published papers, I don’t have the confidence in supervising someone how to write an actual Honours paper. P14 Clinician*  *I think working as part of a team, even the supervision is really a good idea, because I had to take some time off for part of the placements, so that was a bit of a challenge of course, but then actually working in partnership with a co-supervisor [Health Service Research Fellow], I think that alleviated that and somebody who knows the project as well… The supervision for the students, so it was really close supervision in terms of we'd catch up with them both during the course of their placement perhaps and like I said, where I've had to be away, she [Research Fellow] sort of then overtook that role as well to supervise the students. P7* *Clinician*  *We realised that it was missing an opportunity and I guess when I moved into a different job, my line manager who was [Health Service Research Fellow] said well, why aren't you going to be on the supervisor? Facilitator: You mentioned that [Health Service Research Fellow] provided input to say why aren't you a supervisor, was that important, the support there? Interviewee: Oh, 100 per cent. Knowing that she was a fan of mine and strong advocate for me and so she could see an opportunity to I guess gain a publication, gain knowledge and for [our health service] to gain more I guess out of the relationship Facilitator: If she hadn't have done that, you would have stayed on the - just the reference group? Interviewee: I think so probably. Facilitator: Even though it's your idea for the project that you put up? Interviewee: Yeah, well, that's kind of when she started asking that question. She's like but isn't it - it's like this is all your idea and all your stuff, how come you're not getting an authorship? And then we had that conversation and followed from there. P5 Clinician*  *[Student] had to finish data collection early because he had a deadline and we had more data coming in, that we collected after that. Interviewer: So who’s managing that data collection? Participant: We’ll have to do it, between me and [Health Service Research Fellow] I guess. P1 Clinician* |
|  | Barrier | Power imbalance between health service clinician and university academic supervisors | I feel a sense that we are representing the health service within [a] project. You could easily get swayed by that power imbalance if you are just a small clinician who feels perhaps lacking in confidence in a certain area like research. P5 Clinician  I guess we’re always going to come up with big personalities and that’s fine, but you know we want to also guard the health services’ interests. Yeah, I think that’s all I’m saying. Probably for nuts and bolts it is just that we should have a level playing field. P2 Clinician  *Then when it came to the CRA, the collaborative research agreement, the first thing we noticed was really wrong was that it said all the background IP belonged to the students... So we had a meeting with the university senior staff member and the other staff member... It would have been great to have additional health service support in that meeting, because essentially what was communicated was that, look you might start a study, but you may never continue on the end of it. …They were suggesting that look, thanks for the idea but we’ll take it from here kind of thing. I was like, no, there’s been a significant amount of our time, both personal time and clinical health work time, that has gone into refining all of the opportunities and possibilities down to this particular concept. Eventually we got the CRA revised that we shared 50:50 IP. P2 Clinician*  Antithesis - Respect for complementary skills:  *We both respected each other’s strengths I think because they had more research experience and I had more of the clinical topic research experience so that worked well. P8 Clinician* |
| D. Reflecting and evaluating | | | |
|  | Facilitator | Regular review concurrent with student placement allowing intervention as required | *I was happy to jump in and take over the design of the survey when I realised it is a difficult task and requires maybe a little bit more specialised knowledge, and I was able to provide that knowledge and I had no problem with that – I felt that was reasonable. P1* *Clinician*  *I just realised at some point that I no longer was really a part of this project. P4 Clinician* |
