# Supplementary material for: The health service perspective on determinants of success in allied health student research project collaborations: a qualitative study guided by the Consolidated Framework for Implementation Research
Source: BMC Health Serv Res. 2024 Jan 27;24:143. doi: 10.1186/s12913-024-10599-8 (PMC10821208; doi:10.1186/s12913-024-10599-8)
Supplement: Supplementary file 3 — Additional file 3. Occurrence of facilitators and barriers to collaborative student research projects by CFIR domain. [file 12913_2024_10599_MOESM3_ESM.docx]

Additional file 3 – Occurrence of facilitators and barriers to collaborative student research projects by CFIR domain

| **CFIR domains and constructs** | **Barriers** | **Number** | **Facilitators** | **Number** |
| --- | --- | --- | --- | --- |
| **I. Innovation Characteristics**  *The act of health service-employed allied health professionals supervising or co-supervising students on a clinically relevant research project undertaken as part of the students’ professional degree qualification program* | | | | |
| 1. Innovation source |  |  | External (EOI circulated from universities)/Internal (decision for involvement made at individual clinician level) | 18 |
| C. Relative Advantage | More efficient to conduct in-house | 3 | Access to student labour, equipment and/or academic research skill | 12 |
| D. Adaptability^2^ | Project altered from clinician’s original idea | 2 | Opportunity to determine project topic  Flexibility of clinician role in project^1^ | 18  3 |
| E. Trialability^2^ |  |  | Discrete commitment enabling trial for further future use^1^ | 3 |
| F. Complexity^2^ | Inflexible university timeframes  Challenges managing ethical approval processes  Team changes over time | 2  6  2 |  |  |
| H. Cost^2^ | Weight of workload  Staff personal time for completion | 12  3 |  |  |
| **II. Outer Setting**  *The external social and political context including Australian and Queensland government policies and in particular, aspects related to universities in the local area offering allied health degree programs* | | | | |
| A. Needs and Resources of Students^2^ | Unengaged students  Burden of university reporting requirements for supervisors^1^ | 5  3 | High achieving/capable students  Pressure of student need ensures progression^1^ | 11  4 |
| B. Cosmopolitanism^2^ |  |  | Invitation from universities  Existing relationships between academics/universities and health service clinicians/departments^1^ | 18  9 |
| **III. Inner Setting**  *The employees, departments, systems, policies and resources of a tertiary hospital and health service located in south-east Queensland* | | | | |
| B. Networks & Communications | Lack of feedback and communication after completion of student placement period | 5 | Regular meetings (including technology facilitated) | 8 |
| C. Culture |  |  | Research valued in department^1^  Awareness of previous research student project in department^1^ | 4  9 |
| D. Implementation climate  D2. Compatibility |  |  | Meeting role requirements^1^ (for research and student supervision)  Familiarity with supervising students through clinical supervision^1^  Recognition of role of health system in training future clinical workforce^1^ | 4  5  7 |
| E. Readiness for Implementation |  |  | Accessibility of research fellows within health service | 8 |
| **IV. Characteristics of Individuals**  *Those of the people employed within the inner setting, specifically, allied health professionals and specialist research staff* | | | | |
| A. Knowledge & Beliefs about the Innovation | Unrealistic expectations of student ability^1^ | 3 | Provides a supported mechanism for conduct of research that would otherwise not progress^1^  Enables research capacity building for the health service workforce (clinicians, future clinicians)^1^  Mechanism for developing research collaborations^1^ | 10  5  5 |
| E. Other Personal Attributes |  |  | Enjoyment of mentoring/working with students^1^  Enjoyment of research and wanting to motivate others in research^1^ | 6  6 |
| **IV. Process** | *The process of conducting the collaborative student research project (the innovation) in its entirety from planning, engaging individuals within the inner and outer settings, executing the project and its evaluation* | | | |
| A. Planning^2^ |  |  | Clear role definitions | 4 |
| 1. Engaging (key stakeholders)   B3. Champions |  |  | Key stakeholders: Personal decision on involvement  Champions: Research fellow involvement | 18  5 |
| C. Executing | Power imbalance between health service clinician and university academic supervisors^1^ | 2 | Health service research fellow support during project  Clinician interest in topic  Value brought by clinical expertise | 13/18^3^  16  14 |
| D. Reflecting and evaluating |  |  | Regular review concurrent with student placement allowing intervention as required | 5 |

Quantification made by number of projects in which participants reported factors occurring, except where annotated. ^1^Quantified by number of participants. ^2^Constructs identified as determinants during data collection and/or analysis. ^3^13/18 projects described by clinician participants - research fellows described additional student projects in which they had supported clinicians.
